# Ebola virus disease: from epidemiology to prophylaxis

**DOI:** 10.1186/s40779-015-0035-4

**Published:** 2015-03-10

**Authors:** Wen Bin Liu, Zi Xiong Li, Yan Du, Guang Wen Cao

**Affiliations:** Department of Epidemiology, Second Military Medical University, 800 Xiangyin Road, Shanghai, 200433 China

**Keywords:** Ebola virus disease, Epidemiology, Outbreak, Virology, Prophylaxis

## Abstract

The outbreak of Ebola virus disease (EVD) continues to spread through West Africa. Since the first report of EVD in March 2014, the number of cases has increased rapidly, with the fatality rate of >50%. The most prevalent Ebola virus belongs to the species of *Zaire ebolavirus*, with a fatality rate as high as 90%. Although there were cases introduced into other continents, Africa is the endemic area where fruit bats and apes are suspected to be Ebola virus carriers. The virus might be transmitted from the host animals to humans if humans consume raw or not fully cooked and contaminated meats. However, human-to-human transmission *via* close contact is the major route of current outbreaks. EVD can occur during any season and affect people of any race and age group. Direct contact with body fluids of EVD patients or living in contaminated environments greatly increases the risk of being infected. Transmission *via* aerosol less likely, but transmission *via* virus-containing droplets is possible in humans. Thus, health care providers are facing danger of getting Ebola virus infection. To date, vaccines, drugs and/or therapies to prevent Ebola virus infection or treat EVD are limited. Medical workers should follow the current standard prophylactic procedures. The military can orchestrate efficient care to mass EVD patients. Although it is necessary to speed up the pace of developing effective vaccine and therapeutics for the prevention and treatment of EVD, public health prevention and management should be important issue at present to control the spread of this disease cost-effectively.

## Introduction

Ebola virus disease (EVD), formerly known as Ebola haemorrhagic fever (EHF), is an acute, severe and fatal disease in humans. In February 2014, the first case of EVD occurred in Guinea, followed by a rapid growth of the EVD epidemic within the next nine months in West Africa. According to the World Health Organization (WHO) update on January 21 2015, EVD had infected 828 medical personnel and caused 499 deaths. By January 23 2015, a total of 21,832 cases or suspected cases, and 8,690 deaths have been reported in Guinea, Liberia, Sierra Leone, Nigeria, Senegal, Mali and elsewhere [1]. Outside of West Africa, imported EVD patients in the United States and Spain caused worldwide panic. A model of Ebola transmission, which was developed based on the incidence data from Liberia, predicted that the epidemic of EVD might continue in early 2015 [2]. Thus, this outbreak has become a global health problem and a deadly threat to humans [3].

Ebola virus is the pathogen of EVD, which is mainly endemic in West Africa. Ebola virus is named after a small river in Zaire (now the Democratic Republic of the Congo, DRC) where the first EVD outbreak occurred in 1976 [4]. Ebola virus is characterized by high lethality, high infectivity, and lack of effective treatment or prophylaxis. Considering the possibility of imported infection and the use for biological terrorism, Ebola virus becomes not only a public health problem to Africa, but also a worldwide bio-threat. This pathogen is listed as a Category A Bioterrorism Agent by the U.S. Centers for Disease Control and Prevention (CDC) [5].

A number of investigational treatments for this fatal disease have been conducted. The main directions of prophylaxis and treatments focus on the development of vaccines, antibody therapies, and antiviral drugs [6]. The promising candidates of these strategies are as follows: Two vectored vaccines, which are based on replication-incompetent chimpanzee adenovirus 3 (cAd3) and replication-competent vesicular stomatitis virus (VSV), have demonstrated 100% protection in non-human primates for 4 to 5 weeks and are undergoing the phase 1 trials [7,8]. Zmap, a combination of 3 monoclonal antibodies, can reverse advanced disease and lead to full recovery in primates under laboratory conditions [9]. Brincidofovir and TKM-Ebola, agents with antiviral activity against EBOV *in vitro*, can be used for antiviral therapy and are also amenable for mass production [6]. Although all of these candidate therapies have enormous potential in clinical applications, most are in the early phase of development, and authorized only for emergency use.

The primary therapeutic strategy for EVD is still symptomatic and/or supportive treatments. The main strategies to control the EVD outbreak have been mainly public health endeavors focusing on epidemiological surveillance, contact tracing, and quarantine [2]. Here, we briefly reviewed the epidemiology, virology, and infectious process of EVD, especially the infectious process and the epidemic characteristics.

## Review

### Epidemical characteristics

#### Epidemic history

EVD is not an immunogenic disease with specific natural seasonality, and it can spread throughout the year as suggested by the historical data of Ebola epidemic outbreaks (Table 1). In 1976, the first reported EVD outbreaks occurred in Sudan [10] and DRC [4]. *Sudan ebolavirus* (SUDV) and *Zaire ebolavirus* (EBOV), which caused these two epidemics, respectively, were the first two human-pathogenic Ebolavirus species isolated. Within a long period, only a small outbreak was reported in 1979, which occurred in the same area as the 1976 Sudan epidemic [11]. From 1994 to 1997, several EVD outbreaks were reported in Gabon and Zaire [12], and another human-pathogenic Ebolavirus, *Taï Forest ebolavirus* (TAFV), was isolated from a single case during this period [13]. Since 2000, the number of the EVD outbreaks has increased in the African continent [14], making the EVD epidemic a major public health concern in Africa. While EBOV and SUDV were responsible for almost all, Bundibugyo virus (BDBV), which first emerged in 2007 in Uganda [15], and then reemerged in 2012 in DRC [16], was also able to cause an epidemic.

**Table 1:** **Epidemic outbreaks of Ebola virus disease in Africa, 1976 to 2014**

**Year**	**Starting month**	**Area**	**Species**	**Cases, n**	**Deaths, n**	**Fatality**	**Ref.**
1976	June	South Sudan	SUDV	284	151	53.2%	[10]
1976	September	DRC	EBOV	318	280	88.1%	[4]
1979	October	South Sudan	SUDV	34	22	64.7%	[11]
1994	June	Gabon	EBOV	52	31	59.6%	[12]
1995	April	DRC^a^	EBOV	315	250	79.4%	[12]
1996	February	Gabon	EBOV	60	45	75.0%	[12]
2000	August	Uganda	SUDV	425	224	52.7%	[17]
2001	October	DRC/Gabon	EBOV	122	96	78.7%	[18]
2002	December	DRC	EBOV	143	128	89.5%	[19]
2007^b^	April	DRC	EBOV	264	187	70.8%	[20]
2007	December	Uganda	BDBV	149	37	24.8%	[15]
2012	November	Uganda	SUDV	14	7	50.0%	[21]
2012	June	DRC	BDBV	52	25	48.1%	[16]
2014^a,b^	March	West Africa	EBOV	19065	7388	38.8%	[20]

EBOV: Zaire ebolavirus; SUDV, Sudan ebolavirus; BDBV, ebolavirus; DRC, The Democratic Republic of the Congo.

^a^Case counts updated December 17, 2014.

^b^Data source: http://www.cdc.gov/vhf/ebola/outbreaks/history/distribution-map [20].

#### Geographical distribution

So far, EVD is mainly endemic to the African continent, especially in West Africa. Other countries, such as the United States, Thailand, United Kingdom, Canada, and Spain had sporadic and possibly imported Ebola cases [22]. The natural environment of the African continent provides a favorable condition for the survival of Ebola virus. First, the natural and alternate hosts of Ebola virus such as fruit bats, apes, and monkeys are widely distributed in Africa. Second, according to the historical data, EVD mainly distributes between 10° north and south of the equator, with the temperature that benefits Ebola virus survival throughout the year.

#### Demographic distribution

Ebola virus can infect people at any age group. In some areas, about 80% of the EVD cases were in adults between 21 and 60 years old [23], possibly because most people at this age range are more likely to be physically active (i.e. hunting for food) and therefore have more opportunities of being exposed to the pathogen. The fatality rate of EVD for the age group of 21 years and under is significantly lower than that in the age group of 45 years and older (57% *vs.* 94%, *P* < 0.05) [24]. Due to the ease of transmission from human-to-human *via* close contact or possibly *via* droplets by sneezing and coughing of patients, medical workers belong to a high-risk population. Some studies have found that the high-risk occupations for EVD are nurses, aid nurses, physicians, laboratory technicians, and physician assistants. Accordingly, hospitals, clinics, and Ebola treatment units are fertile for ebolavirus transmission [25]. Different genetic backgrounds of affected populations may relate to different susceptibility to EVD [26], future mechanistic studies should investigate the role of genetic factors on the development and progression of EVD.

### Virology

#### Family members and viral genome

Ebolavirus belongs to the *Filoviridae* family and contains 5 members: EBOV, SUDV, TAFV, BDBV and *Reston virus* (RESTV) [27]. With the exception of RESTV, which is only found in the Philippines, all other four members are the causative agents of EVD that are endemic to West Africa. EBOV is the most virulent species, followed by SUDV. The RESTV is the mildest among these 5 species, and only causes disease in non-human primates [28].

EBOV is a non-segmented negative-sense single-stranded RNA virus with filamentous particles, a morphological characteristic of all Filoviruses [5]. EBOV particles are the same in width (80 nm) but vary in length (up to 1400 nm), which can be coiled, toroid or branched. The EBOV genome is approximately 1.5 kb in length and contains seven genes encoding nucleoprotein, virion protein (VP) 35, VP40, glycoprotein (GP), VP30, VP24, and RNA-dependent polymerase [22].

The unstable RNA genome of EBOV leads to a high mutation rate [14]. Phylogenetic analysis based on sequencing technology is reliable for epidemiological surveillance. In 2014 EVD outbreak, investigators provide timely information about the origins and the transmission routes of EBOV, which was crucial in recognizing the pathogens at the initial period of the outbreak [29]. In the previous outbreaks, the mutation rates of EBOV were relatively rapid, ranging from 1.97 × 10^−4^ to 10.7 × 10^−4^ nucleotide substitutions/site/years in human populations. But the low diversity within the EBOV variants revealed that the high mutation rates did not facilitate the EBOV evolution process, which was believed to undergo a bottleneck period [30]. However, the EBOV mutation rates in the 2014 outbreak strains were as high as 2 × 10^−3^ nucleotide substitutions/site/years [29], which was on average 2 to 3 times higher than that observed in the previous epidemics. The high viral mutation rate of the 2014 EVD outbreak strains implied the potential of a rapid evolution. The mutant strains of EBOV can be generated with different virulence, infectivity, tissue tropism, and the capability of immunosuppression. The viral variants might be selected by specific pressures, including the immune response, treatment, and the condition of infection. As the genetic surveillance data suggested, the number of EBOV lineages increased along with the increased number of affected populations [29]. The repeated passage of EBOV in humans can allow the viruses to accumulate mutations with phenotypic impact. Therefore, EBOV may become more transmissible or lethal, highlighting the importance of genetic surveillance for the viruses.

#### Immunosuppression function

EBOV is immunosuppressive, which is a major obstacle for the development of effective vaccination. Deceased EVD patients usually failed to trigger effective immune responses after infection; whereas effective immune responses are frequently sustained in survivors. The roles of EBOV-encoded proteins in the suppression of host immune responses have been extensively investigated in the past decades. Major advances have been reported on three aspects. First, VP35 and VP24 can function as important virulence determinants that allow EBOV to evade the antiviral effect of type I interferon (IFN). VP35 has been demonstrated to function as an IFN antagonist that inhibits type I IFN responses in Ebola virus-infected cells [31]. VP24, a structural protein associated with the membrane, can block IFN signaling by impairing nuclear accumulation of tyrosine-phosphorylated STAT1 [32]. Second, EBOV secretory glycoprotein (sGP), a primary product of the GP gene, enables the virus to escape from neutrophils and avoid being wiped out by immune system. sGP, is soluble and can be secreted in large quantities by infected cells and inhibit the early activation of neutrophils by binding to CD16b leading to suppression of anti-viral innate immunity. sGP also facilitates EBOV replication [33]. This partially explains why EBOV can spread rapidly in the host while evading immune eradication. Lastly, sGP can serve as a decoy for GP by binding to neutralizing antibodies produced by the host. Neutralizing antibodies, which defend a cell from being infected by viruses through binding to antigen and neutralizing its biological effects, are not usually detected even in the convalescent survivors. Furthermore, there is evidence of shared neutralizing epitopes on GP and sGP molecules and sGP is believed to substitute GP from biding neutralizing antibodies and consequently reducing antibody titer [34].

#### Stability and viability

In blood and or other body fluids, or on contaminated surfaces, EBOV can survive for hours at room temperature (20°C−25°C), and for weeks at low temperature (4°C) [17]. EBOV is only moderately heat resistance and can be inactivated by heat treatment (>60°C) for at least 1 hour. EBOV is also sensitive to ultraviolet light, gamma rays, and many chemical reagents, including ether, peracetic acid, sodium hypochlorite, and formaldehyde [35].

### Infectious process

#### Sources of infection

Patients at the contagious stage are the main sources of human-to-human transmission. The viruses can exist in body fluids such as blood, semen, and genital secretions as well as the skin of contagious patients [36,37]. The incubation period is up to 21 days, which is the epidemiological basis for quarantine.

Fever and other EVD symptoms such as headache, fatigue, and diarrhea often appear at the earlier contagious stages and before significant alterations of the laboratory indexes, allowing for timely identification of infected patients [38]. However, the initial clinical signs of EVD lack specificity. Therefore, laboratory test are indispensible for a confirmative diagnosis. Polymerase-chain-reaction (PCR) test for EBOV nucleic acid and the detection for the viral antigen in the blood can become reliably positive from 2 to 16 days after the onset of symptoms. Immunoglobulin M (IgM) can be detected as early as 2 days after the onset and immunoglobulin G (IgG) usually appears between 6 and 18 days after the appearance of the clinical signs [39]. The persistence of IgM and rising IgG titers are important markers to diagnose EBOV infection. In successive paired serum samples, a decrease of IgM titer or an increase (more than four-fold) of IgG titer, or both can characterize a recent infection [22]. IgM usually disappears within 30 to 168 days after infection, while IgG can persist for years [39].

The cadaver is also an important source of infection. The traditional burial rituals in Africa usually involve close contact with the corpse, which facilitates the spread of EBOV. Epidemiological data reveal that 68% of the infected cases in Guinea during the 2014 EVD outbreak were associated with funerals [40].

Convalescent patients may have subclinical EBOV. Viral RNA can be detected for up to 1 month in vaginal, rectal, and conjunctival secretions and up to 3 months in semen samples after the disease onset [36], indicating the persistence of EBOV in convalescent patients. Although sexual transmission is also possible, there is no evidence supporting the transmission between convalescent patients and their spouses [36].

Asymptomatic carriers of EBOV, who do not become ill after physical contact with EVD patients, play a very limited role in the EVD outbreak. Invaded viruses are often quickly wiped out by the efficient immune responses, leading to a low viral load and short-time inflammatory reaction that disappears in 2–3 days without subsequent tissue and organ injury. It is commonly believed that an asymptomatic carrier is not infectious. This supposition is supported by the field experience in West Africa [38]. However, the first case of asymptomatic carrier was reported in 1996 from the outbreak in northern Gabon, and the analysis of nucleoproteins isolated from the carrier and EVD patients revealed no differences [41]. In addition, the occult EVD infection usually occurs in those racial groups living in the tropical rainforest regions. The prevalence of occult EVD infection is as high as 10% in the rainforest areas in the Guinea - Congo Basin [42]. These data suggest that the existence of asymptomatic carriers is possibly because of the racial and population differences, and/or diverse genetic background, rather than infection with low-virulence mutants of EBOV.

EVD is a typical zoonotic disease, but the wild reservoir of EBOV is still unclear. Non-human primates, like apes or monkeys, have long been considered as important sources of infection to humans; however, these primates might not be original reservoir species because they could be killed by this infection [28]. Since the first EVD outbreak in 1976, many studies have tried to identify the natural reservoirs. An experimental study inoculated African plants and animals with EBOV in the laboratory, and detected high titers of the virus both in asymptomatic fruit bats and insectivorous bats [43]. Although no solid link between EBOV and bats is established, this evidence provides a promising clue of potential candidates. During the 2001 to 2003 Ebola outbreaks in humans and apes in Gabon and DRC more than a thousand animals were collected from the epidemic areas for testing. EBOV RNA and antibodies were found in three species of fruit bats with asymptomatic infections [44]. It is a milestone to find natural reservoir species with asymptomatic or subclinical infection. The distribution of habitats, activity range, and natural characteristics of the reservoir animals, may explain the sporadic nature and periodicity of EVD outbreaks, and changes in these factors may increase the opportunity of animal-to-human transmission of EBOV [22].

#### Route of transmission

Close contact is the most important route of EBOV transmission. EBOV can survive in body fluids of patients or cadavers for days, and invade into recipients’ bodies *via* mucous membranes and/or broken skin. Besides the body fluids and skin of EVD patients or cadavers, the virus also can spread *via* recently contaminated items like clothes [41,45,46]. The first patients of the past EVD outbreaks often had a history of contact, such as eating or handling ill or dead EBOV-infected non-human primates or other mammals, while most of the subsequent cases were associated with hospital-mediated dissemination and intra-familial transmission [47].

Oral transmission is another important route in Africa. Sick or virus-carrying animals can be easily hunted for food. Consumption of bushmeat that is not cooked thoroughly is a common way of exposure to infected animals. Fruit bat is possible candidate of the EBOV reservoir animals [44,48]. Fruit bats mainly distribute throughout equatorial Africa and can spread EBOV during hunting [49]. Although EBOV can be inactivated by heat, consumption of freshly killed animals is still very dangerous. The high lethality of EBOV invading through oral transmission was demonstrated by non-human primate experiments [50]. The putative first human victims of the outbreak in Gabon [51] and in DRC [49] were associated with consumption of chimpanzees and fruit bats, respectively, which led to the subsequent human-to-human transmission. Notably, EBOV was discovered in pigs in the Philippines in July 2008 [52], highlighting the possibility of the wide range of potential animal hosts, especially domestic animals.

Nosocomial infection also plays an important role in EBOV transmission, especially in regions with inadequate hygienic resources. The 1976 outbreaks of Ebola virus in Sudan and Zaire were due to the reuse of contaminated needles [45,46]. The incubation period of EVD is on average 6.3 days for cases with injection exposure, and 9.5 days for those infected through contact transmission [22]. Healthcare providers are usually at high risk of being exposed to contaminated needles, samples, corpses, and/or biological materials. Therefore, it is vital to operate with standard protective procedures and equipments.

Airborne transmission of EBOV is still in debate [53]. According to the previous EVD outbreaks, infection does not occur without direct physical contact, even when in the presence of EVD patients in confined spaces, suggesting EVD is not transmitted *via* airborne routes. Given the low viral concentration in the lungs, it would be difficult for EBOV to spread through airborne transmission [54]. Only pigs with EVD, which accumulate large numbers of EBOV in their lungs, can spread virus to the nonhuman primates through airborne transmission [55]. However, EBOV may survive in the saliva of severe cases and infect people who are splashed with contaminated droplets. This suggests a risk of airborne EBOV transmission between severe patients and the medical personnel caring them.

#### Susceptibility

All human races are susceptible to EBOV, but the outcome of EVD depends on the transmission route, viral load, age, and probably genetic background. The case fatality rate of patients infected by injection is much higher than those by contact exposure [22]. The case fatality rate of patients with high viral load (>10^7^ copies/ml) is approximately 3 times higher than that of those with low viral load (<10^4^ copies/ml). Patients over 45 years of age have a higher case fatality rate than those under the age of 21 [56]. Results from the experiment of a novel EBOV infection mouse model suggested that host genetic background played a significant role in determining the prognosis of EVD [26]. Although infected with mouse-adapted EBOV under the same conditions, different genetic backgrounds had different outcomes ranging from complete resistance to lethal disease. The evidence of animal experiments is consistent with epidemiological data (high percentage of asymptomatic carriers in the rainforest racial group) and clinical characteristics (significant differences among the human disease spectrum of the infected patients), suggesting that genetic diversity plays a role in EVD pathogenesis and/or resistance.

### Prevention and control

Since there are no specific medications broadly available to cure EVD, the most important thing is to prevent susceptible people from the infection and restrict spreading. This requires the government, public health facilities, medical units, and individuals to make concerted efforts. Military forces are indispensable when local public health systems are overwhelmed or in the case where the EVD epidemic goes out of control.

**Figure 1:**
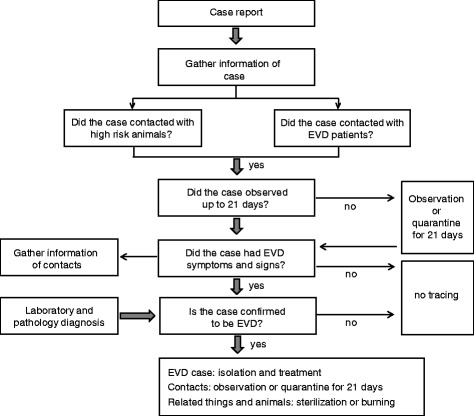
**Case confirmation: Ebola virus disease.**

Sufficient political support from the government is crucial for the prevention and control of the Ebola epidemic. First, a strong public health infrastructure and medical reserve should be established and improved. Most severe EVD epidemics occurred in areas where the health systems were overwhelmed or failed to identify and isolate the infection cases in a timely fashion [57]. Second, contact tracing and quarantine policies should be strengthened. Briefly, persons of a close contact with EVD patient should be monitored if the related symptoms are present within 21 days. New cases should be identified and isolated quickly, and the cycle should be repeated until no patient emerges. A well-designed and appropriately operated disease surveillance system should be in place when a suspected case is reported (Figure 1). Third, considering the absence of effective treatment and the high case fatality, it is reasonable to circumvent research ethics and authorize the promising experimental vaccines or drugs for emergency use. Lastly, epidemiological and clinical data of Ebola should be collected vigorously and systematically in the endemic areas. This is the basis of epidemiological or genetic surveillance and relies on the coordination of public organizations and agencies [29].

The Ebola treatment units encounter two challenging tasks: to optimize the use of available medical resources and to avoid nosocomial infections. Clinical assessment could triage EVD patients into three categories: those who are able to provide self-care, not in shock, just hypovolemic; those who are unable to provide self-care, have hypovolemic, but not in shock; and those in shock with organ failure [58]. This grouping strategy can help save medical resources and achieve maximum effect on disease control. Epidemiological data have demonstrated that health care providers are facing an increased occupational risk of Ebola infection [25]. All hospitals, laboratories and other health care facilities should follow the established standard procedure when treating patients as well as collecting and handling patients’ specimens [59]. In addition, the adequate use of personal protective equipment recommended by the U.S. CDC is an obligation for health care providers to ensure that no skin is exposed to any possible source of infection.

Individuals in EVD endemic areas should take prophylactic measures to avoid any possible risk of getting infected. Certain lifestyle changes such as regular hand washing, avoid gatherings, and changes in the traditional rituals may significantly reduce the risk of Ebola transmission [57]. Contact with and the consumption of wild animals that may be the reservoirs of EBOV, like bats and primates, should be avoided. Contaminated objects of patients, like clothes and towels, should be also disinfected or burned without delay.

Military forces should respond in a timely manner to EVD outbreak if it becomes an increasing threat to the international society. In the most affected countries, the EVD epidemic not only caused great casualties, but also brought the collapse of economic and health infrastructures. Displacement of masses of refugees can spread the disease rapidly to nearby developing countries and even developed countries, leading to an exponential increase of infected cases and death tolls. Thus, effective control of EVD outbreak is not only an emergency issue to medical community, but also a health protection mission to military force. With the quick response capability and well-organized deployment, the military experience has demonstrated how to orchestrate efficient medical care to mass casualties in an austere environment within a limited period. Besides, military forces can ensure effective evacuations to keep health workers flowing, and build temporary isolated units and treatment facilities like the mobile army surgical hospital (MASH). During the 2014 West Africa EVD outbreak, many international volunteers and organizations took care of thousands of EVD patients in the epidemic areas. But due to inadequate medical resources and lack of logistical support, those organizations were overwhelmed and failed to stop the spread of EBOV. In September 2014, both Médecins Sans Frontières (MSF) and the European Community Humanitarian Aid Office (ECHO) who had played a major role in the international effort in controlling outbreak asked international military medical assistance [60]. In the meantime, the United Nations Security Council declared Ebola a global security threat, and created the Mission for Ebola Emergency Response (UNMEER), which was the first health mission in its history [61]. The US, UK, Germany, and China responded promptly. The US and UK sent troops to established isolation units and treatment centers in Liberia and Sierra Leone, respectively. Approximately 5,000 German troops volunteered to work in the West Africa [62]. China also dispatched several testing and medical teams including military health units to set up hospitals in Sierra Leone and to establish or upgrade lab screening systems, and participate in the treatment of EVD [63].

## Conclusion

As the EVD outbreak is continuously raging in West Africa, it is a daunting task to control the outbreak. Since the first Ebola outbreak in 1976, there have been limited measures to reduce the high mortality. Future efforts need to focus on developing effective vaccines, drugs and therapies. More studies are needed to confirm the pathophysiology of the infection in order to identify new targets for medical intervention. Although it is necessary to speed up the pace of developing effective vaccine and therapeutics for the prevention and treatment of EVD, public health prophylaxis is the most important issue at present to control the spread of this disease cost-effectively. International cooperation, especially the cooperation between medical and military systems is critical to prevent and control the epidemic of EVD.

## Abbreviations

BDBV: Bundibugyo ebolavirus
cAd3: Replication-incompetent chimpanzee adenovirus
CDC: Centers for Disease Control and Prevention
DRC: Democratic Republic of the Congo
EBOV: Zaire ebolavirus
ECHO: European Community Humanitarian Aid Office
EHF: Ebola haemorrhagic fever
EVD: Ebola virus disease
GP: Glycoprotein
IFN: Interferon
IgG: Immunoglobulin G
IgM: Immunoglobulin M
MASH: Mobile army surgical hospital
MSF: Médecins Sans Frontières
NAb: Neutralizing antibody
PCR: Blood polymerase-chain-reaction
RESTV: Reston ebolavirus
sGP: Secretory glycoprotein
SUDV: Sudan ebolavirus
TAFV: Taï Forest ebolavirus
VP: Virion
VSV: Replication-competent vesicular stomatitis virus
WHO: World Health Organization
